# Clinical characteristics of patients requiring emergency hospitalization due to immune-related adverse events: a retrospective study

**DOI:** 10.1186/s40780-024-00400-7

**Published:** 2024-12-18

**Authors:** Tatsuki Ikeda, Satoru Nihei, Kazuki Saito, Junichi Asaka, Kenzo Kudo

**Affiliations:** 1 Department of Pharmacy, Iwate Medical University Hospital, 2-1-1 Idaidouri, Yahaba-Cho, Iwate, 028-3609 Japan; 2 Department of Clinical Pharmaceutics and Pharmacy Practice, School of Pharmacy, Iwate Medical University, 2-1-1 Idaidouri, Yahaba-Cho, Iwate, 028-3609 Japan

**Keywords:** Immune checkpoint inhibitors, Retrospective study, Emergency hospitalization, Immune-related adverse events

## Abstract

**Background:**

Immune checkpoint inhibitors (ICIs) have revolutionized cancer treatment, offering hope for various malignancies by enhancing the immune response against tumors. However, ICIs are associated with unique immune-related adverse events (irAEs), which differ significantly from conventional chemotherapy-induced toxicities. These irAEs, which affect more than 70% of patients and often escalate to severe grades, present substantial clinical management challenges and frequently necessitate emergency hospitalization. Therefore, this study aimed to investigate the clinical characteristics of patients requiring emergency hospitalization due to irAEs during ICI therapy to enhance understanding and improve management strategies.

**Methods:**

This retrospective study evaluated patients who received ICIs at Iwate Medical University Hospital between August 1, 2016, and December 31, 2022, and required emergency hospitalization due to irAEs. Clinical data were extracted from the medical records, including patient demographics, presenting complaints, time from ICI initiation to hospitalization, irAE diagnoses, and treatment outcomes. The Spearman rank correlation coefficient was used to analyze the associations between the chief complaints and irAE diagnoses.

**Results:**

Of 1009 ICI-treated patients, 96 required emergency hospitalization for irAEs. The cohort's mean age was 73 years, with 75.0% of patients being male. Among patients who required emergency hospitalization, a high proportion were undergoing treatment for lung cancer (41.7%). The median hospitalization duration was 87 days. The chief complaints included dyspnea (34.4%) and fatigue (34.4%), with gastrointestinal and respiratory disorders being the most frequent irAEs (35.4%). Significant correlations were observed between dyspnea and respiratory diseases (Rs = 0.66), skin diseases and disorders (Rs = 0.81), pain and musculoskeletal disorders (Rs = 0.59), and diarrhea and gastrointestinal disorders (Rs = 0.49). Corticosteroids were administered to 64.6% of the patients. Despite emergency interventions, 8.3% of patients succumbed to irAEs, while 33.3% resumed ICI therapy after hospitalization.

**Conclusions:**

Emergency hospitalization due to irAEs is a considerable concern in ICI therapy, occurring in 9.5% of treated patients. The high incidence of severe irAEs within the first 3 months of treatment underscores the need for early and vigilant monitoring. This study highlights the importance of recognizing and promptly managing irAEs to improve patient outcomes. Future strategies should focus on developing comprehensive management frameworks and enhancing patient and caregiver education to recognize symptoms that warrant immediate medical attention.

**Supplementary Information:**

The online version contains supplementary material available at 10.1186/s40780-024-00400-7.

## Background

The use of immune checkpoint inhibitors (ICIs) has expanded substantially across various malignancies, establishing their pivotal role in contemporary oncologic therapy [1–3]. Integrating ICIs with conventional cytotoxic agents and molecularly targeted therapies offers synergistic therapeutic options for patients with cancer. Nonetheless, ICIs are associated with immune-related adverse events (irAEs), which differ from conventional toxicities owing to their unpredictable onset and severity, posing substantial clinical management challenges [4–6].

The high incidence of irAEs, affecting more than 70% of patients and frequently escalating to grade 3 or higher, underscores the critical need for effective management strategies [7–9]. These events often necessitate treatment interruption, resulting in diminished quality of life and occasional mortality. Effective management requires prompt intervention beyond routine clinical procedures, including unscheduled visits and emergency department admissions. Furthermore, it is crucial to determine whether these emergencies are identified during routine follow-up visits or as unscheduled emergency presentations. This classification aids in understanding the patterns and urgency of these interventions.

Previous studies have investigated the incidence, types, and general management of irAEs, focusing primarily on emergency room visits triggered by these events [10–13]. However, the clinical characteristics, chief complaints, and outcomes of the patient population in which irAEs require emergency hospitalization have been less well described in the literature [14]. Emergency department visits are documented, but data on the circumstances and management of emergency hospitalizations, which are expected to be more often associated with more serious irAEs, are scarce. Addressing this gap is crucial, as it limits our ability to optimize emergency care protocols and develop prevention strategies aimed at reducing the severity and frequency of these hospitalizations. To clarify the circumstances leading to critical interventions, this study aimed to examine the context in which irAEs leading to emergency hospitalizations are identified, whether during scheduled outpatient visits or emergency presentations. Hence, this study investigated patients requiring emergency hospitalization due to irAEs during ICI therapy to elucidate their clinical characteristics, including presenting complaints, symptomatic profiles, and therapeutic modalities, to enhance the safety profile of ICI therapy.

## Methods

### Ethics statements

This study was approved by the Ethical Review Committee of Iwate Medical University (MH2023-004), and the requirement for informed consent was waived owing to the retrospective nature of the study.

### Study design and population

To investigate patients requiring emergency hospitalization owing to irAEs during ICI therapy, this retrospective study included patients with malignant neoplasms who were administered ICIs at the Iwate Medical University Hospital Outpatient Chemotherapy Center between August 1, 2016, and December 31, 2022, and subsequently required emergency hospitalization due to irAEs. The cutoff date for data inclusion was August 31, 2023, and clinical data were extracted from the medical records.

We reviewed all emergency admissions after at least one ICI cycle by an outpatient chemotherapy provider. Patients with multiple emergency hospitalizations were counted as one. Toxicity was confirmed by examining the patient’s course of hospitalization. For each emergency admission analyzed, patient trends were recorded, including their chief complaint at the time of emergency admission, time from ICI initiation to emergency admission due to irAE, classification of the first emergency admission as routine or non-routine, and diagnosis of irAE. In addition, the severity of the chief complaint at the time of admission was graded using the Common Terminology Criteria for Adverse Events (CTCAE). Details of post-hospitalization treatment, discharge, death, other outcomes, and whether ICI was resumed were recorded. Each outcome was evaluated not only overall, but also separately for scheduled outpatient visits or emergency presentations. Demographic data, such as age, sex, cancer type, and treatment regimen, were extracted for all emergency admissions. Patients were excluded from the study if their admission was unrelated to irAE, elective or planned, if they had not received at least one cycle of ICI treatment prior to emergency admission, or if their medical records lacked sufficient data to confirm irAE. The criteria for emergency hospitalization following a scheduled outpatient visit in this study included only those cases in which the patient had significant subjective symptoms, such as severe fatigue, dyspnea, or gastrointestinal problems before or during the office visit, and was judged by the treating physician to require immediate inpatient management. Patients who requested hospitalization for personal reasons were excluded.

### Statistical analysis

Spearman rank correlation coefficient (Rs) and two-sided *P* values were used to evaluate the association between chief complaints and irAE diagnosis at the time of emergency admission. Treatment after emergency admission and subsequent resumption of ICI were also evaluated using Fisher's direct probability test. A correlation coefficient (Rs) ≥ 0.4 indicated a significant relationship, and statistical significance was defined as *P* < 0.05. Statistical analysis was performed using BellCurve for Excel (Social Research and Information Corporation).

## Results

### Patient demographics

**Fig. 1:**
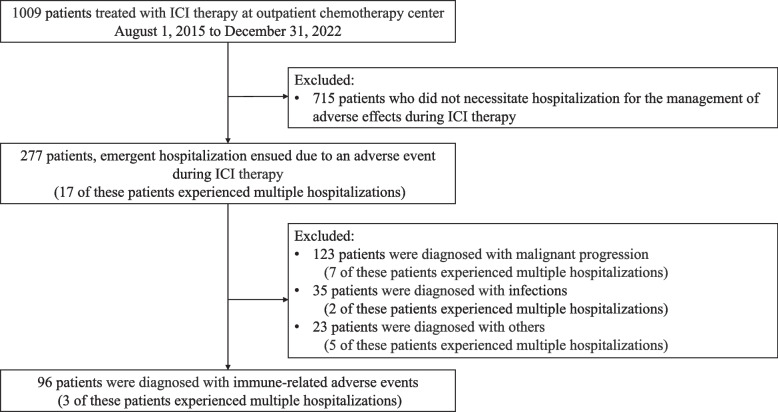
Flowchart of study selection and design

During the study period, 1009 patients received ICI therapy, and 96 emergency hospitalizations were analyzed (Fig. 1). The baseline characteristics of the patients are presented in Table 1. Hospitalized patients’ mean age was 73 years (range, 19–88 years), and 75.0% of the cohort were male. Among the hospitalized patients, a high proportion were undergoing treatment for lung cancer (41.7%), followed by melanoma (17.7%). The treatment regimens included ICI monotherapy in 78.1% of patients and combination therapy in 21.9%. The initial clinical status of scheduled outpatient visits accounted for 52.1% of admissions, while emergency presentations accounted for 47.9%.

**Table 1:** Patient characteristics and initial clinical status (*n* = 96, %)

Age, median (range), y	73 (19–88)
Men	72 (75.0)
Cancer type	
lung cancer	40 (41.7)
Melanoma	17 (17.7)
Renal cell carcinoma	15 (15.6)
Stomach cancer	6 (6.3)
Esophageal cancer	6 (6.3)
Urothelial cancer	3 (3.1)
Head and neck cancer	2 (2.1)
Uterine cancer	2 (2.1)
Liver cancer	1 (1.0)
Breast cancer	1 (1.0)
Colorectal cancer	1 (1.0)
Hodgkin's lymphoma	1 (1.0)
Cancer of unknown primary	1 (1.0)
Treatment regimen	
ICI Monotherapy	75 (78.1)
ICI Combination Chemotherapy	21 (21.9)
Initial response status	
Scheduled outpatient visits	50 (52.1)
Emergency presentations	46 (47.9)

The placement of Table 1 is on page 12, after Patient demographics

*Abbreviations:* *ICI* Immune checkpoint inhibitor, *irAEs* immune-related adverse event

### Clinical characteristics of emergency admissions with irAEs

**Fig. 2:**
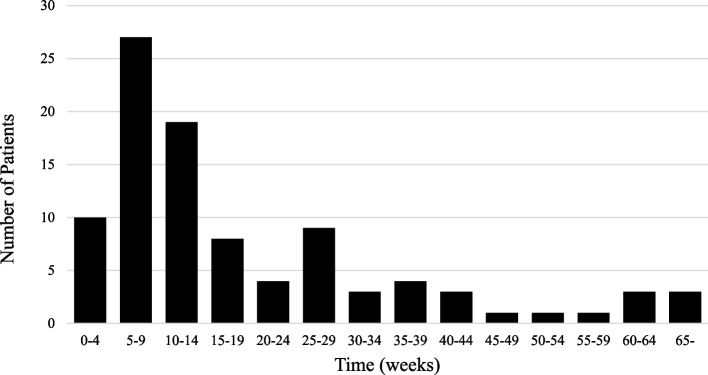
Time from start of ICI treatment to emergency admission due to irAE. Vertical axis; number of patients (n). Horizontal axis; time from start of ICI treatment to emergency admission (weeks)

**Fig. 3:**
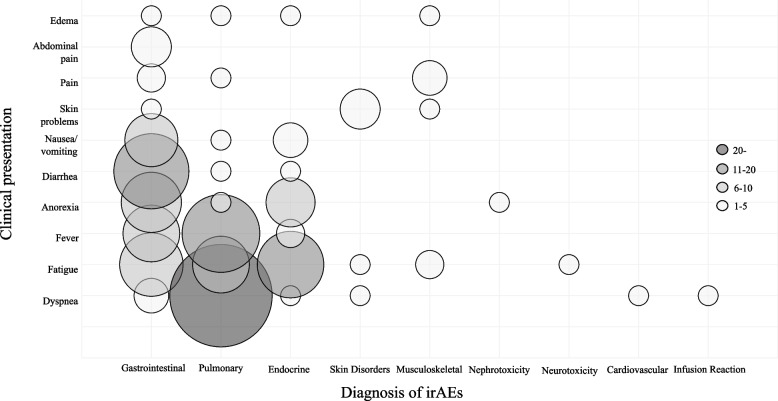
Correlation analysis of chief complaints and diagnoses. The bubble chart shows number of diagnosed irAEs and chief complaint episodes represented by the size and color of the circles. Vertical axis; chief complaint at admission (episodes, with multiple chief complaints). Horizontal axis; diagnosed irAE. Abbreviations: irAEs, immune-related adverse events. Gastrointestinal; Gastrointestinal disorders, Pulmonary; Pulmonary disorders, Endocrine; Endocrine disorders, Musculoskeletal; Musculoskeletal disorders, Nephrotoxicity; Nephrotoxicity disorders, Neurotoxicity; Neurotoxicity disorders, Cardiovascular; Cardiovascular disorders

The median interval from the initiation of ICI treatment to emergency admission was 87 days (range, 8–1006 days), with 27 patients occurring within the first 5–10 weeks, accounting for 28.1% of all patients (Fig. 2). The interval to emergency hospitalization by diagnosis of irAE is shown in Supplementary Materials 1. Table 2 shows the chief complaint at the time of emergency admission and the subsequent irAE diagnosis. A study of treatment schedules in 50 patients with scheduled outpatient visits showed that 25 patients were treated at 2-week intervals, 21 at 3-week intervals, three at 4-week intervals, and one at 6-week intervals. Three patients had multiple hospitalizations due to irAE, all three had two experiences. Two of the three patients were male. two had lung cancer and one had hepatocellular cancer. One patient went through the scheduled outpatient clinic and two were in the emergency department. The diagnoses of irAE were gastrointestinal toxicity, pulmonary toxicity, and endocrine toxicity, respectively. Dyspnea and fatigue were the predominant chief complaints, accounting for 34.4% of admissions. The severity of the chief complaint at admission was assessed using CTCAE and classified into groups of Grade 2 or less and Grade 3 or more. Of the patients analyzed, 67.7% had Grade 2 or less symptoms, while 32.3% had Grade 3 or higher symptoms. Gastrointestinal and respiratory disorders were the most prevalent irAEs (35.4%), followed by endocrine disorders (16.7%); skin and musculoskeletal disorders (4.2%); and renal, neurological, cardiac, and infusion reactions (1.0%). In this study, fatigue and fever were particularly common complaints in patients requiring emergency presentations, with fatigue observed in 39.1% of cases. Figure 3 shows the association between chief complaints and irAE diagnoses. The Spearman rank correlation coefficient (Rs) was calculated for each pair of chief complaints and irAE diagnoses to further investigate these associations. Significant correlations were observed between dyspnea and respiratory diseases (Rs = 0.66), skin diseases and skin disorders (Rs = 0.81), pain and musculoskeletal disorders (Rs = 0.59), and diarrhea and gastrointestinal disorders (Rs = 0.49), all of which were statistically significant (*P* < 0.01). A table detailing the results of the statistical analysis is presented in Supplementary Material 2. The analysis of post-hospitalization treatment vs. ICI therapy did not reach statistical significance with a *P* value of 0.7682.

**Table 2:** Clinical characteristics of Emergency admissions with irAE

	Whole sample (%)	Scheduled outpatient visits (%)	Emergency presentations (%)
*n*	96	50	46
Chief complaints and Clinical presentation			
Fatigue	33 (34.4)	15 (30.0)	18 (39.1)
Grade3-4	7 (7.3)	2 (4.0)	5 (10.9)
Dyspnea	33 (34.4)	20 (40.0)	13 (28.3)
Grade3-4	8 (8.3)	4 (8.0)	4 (8.7)
Fever	25 (26.0)	8 (16.0)	17 (37.0)
Grade3-4	0	0	0
Anorexia	17 (17.7)	7 (14.0)	10 (21.7)
Grade3-4	3 (3.1)	0	3 (6.5)
Diarrhea	16 (16.7)	6 (12.0)	10 (21.7)
Grade3-4	4 (4.2)	2 (4.0)	2 (4.3)
Nausea/vomiting	11 (11.5)	5 (10.0)	6 (13.0)
Grade3-4	3 (3.1)	0	3 (6.5)
Skin problems	6 (6.3)	2 (4.0)	4 (8.7)
Grade3-4	2 (2.1)	2 (4.0)	0
Pain	6 (6.3)	3 (6.0)	3 (6.5)
Grade3-4	0	0	0
Abdominal pain	4 (4.2)	1 (2.0)	3 (6.5)
Grade3-4	1 (1.0)	0	1 (2.2)
edema	4 (4.2)	1 (2.0)	3 (6.5)
Grade3-4	1 (1.0)	0	1 (2.2)
Abnormal laboratory values	23 (24.0)	18 (36.0)	5 (10.9)
Other	10 (10.4)	3 (6.0)	7 (15.2)
Type of irAE			
Gastrointestinal disorders	34 (35.4)	19 (38.0)	15 (32.6)
Pulmonary disorders	34 (35.4)	16 (32.0)	18 (39.1)
Endocrine disorders	16 (16.7)	9 (18.0)	7 (15.2)
Skin disorders	4 (4.2)	2 (4.0)	2 (4.3)
Musculoskeletal disorders	4 (4.2)	2 (4.0)	2 (4.3)
Nephrotoxicity disorders	1 (1.0)	0	1 (2.2)
Neurotoxicity disorders	1 (1.0)	1 (2.0)	0
Cardiovascular disorders	1 (1.0)	1 (2.0)	0
Infusion Reaction	1 (1.0)	0	1 (2.2)

The placement of Table 2 is on page 14, after Clinical characteristics of emergency admissions with irAEs

*Abbreviations*: *irAEs* immune-related adverse event

### Treatment and outcome

Table 3 shows the posthospitalization treatments and outcomes. Corticosteroids were administered to 64.6% of patients, while corticosteroids combined with an additional immunosuppressant were used in 4.2%. Hormone supplementation was employed in 16.7% of patients, and all patients admitted with endocrine disorders received replacement therapy. In the hospitalized cohort, 33.3% resumed ICI therapy, and 55.2% discontinued it. Additionally, 8.3% of patients died of irAEs. Supplementary Material 3 provides detailed information on posthospitalization outcomes.

**Table 3:** Posthospitalization treatments and outcomes

	Whole sample (%)	Scheduled outpatient visits (%)	Emergency presentations (%)
*n*	96	50	46
Treatment			
Steroids	62 (64.6)	33 (66.0)	29 (63.0)
Steroids and immunosuppressive	4 (4.2)	2 (4.0)	2 (4.3)
Hormone supplementation	16 (16.7)	7 (14.0)	9 (19.6)
Outcome			
Resume administration of ICI	32 (33.3)	15 (30.0)	17 (37.0)
Discontinue administration of ICI	53 (55.2)	30 (58.0)	23 (52.2)
Death subsequent to hospitalization	8 (8.3)	4 (8.0)	4 (8.7)
Other	3 (3.1)	2 (4.0)	1 (2.2)

The placement of Table 3 is on page 15, after Treatment and outcome

*Abbreviations*: *ICI* Immune checkpoint inhibitor

## Discussion

Over the 7-year study period, 1009 patients received ICI therapy at our institution, with 96 patients requiring emergency hospitalization due to irAEs, with a hospitalization rate of 9.5%. Moreover, 47.9% of hospitalized patients required unplanned medical facility visits, highlighting the need for interventions beyond standard clinical protocols. Clinical trials of approved ICIs have reported emergency hospitalizations in fewer than 5% of patients [15–17]. In contrast, our study indicated a higher frequency of emergency hospitalizations, suggesting a potentially increased incidence of irAE-related emergencies in real-world settings. These findings underscore the importance of understanding the clinical characteristics of emergency hospitalizations for irAEs and advocating an emergency response framework that ensures continuous monitoring and prompt intervention.

Approximately 40% of the emergency hospitalizations due to irAEs in our study occurred within the first 3 months of treatment, consistent with previous reports showing irAE onset within 2–3 months of ICI administration [18, 19]. Early monitoring of irAE onset and severity is therefore crucial for effective management. In this study, treatment intervals for scheduled hospitalizations ranged from 2, 3, 4, and 6 weeks. While this variation reflects the flexibility of ICI treatment regimens, longer intervals may delay irAE detection and increase the risk of emergency hospitalization, whereas more frequent visits may allow earlier detection and reduce the need for emergency intervention. Thus, patients with long intervals between visits require careful monitoring, including the use of telecommunications. The nearly equal frequency of routine and emergency visits in our study also highlights the limitations of relying solely on routine outpatient visits to manage ICI-related adverse events. Given the unpredictable nature and variable frequencies of irAEs, it is essential to establish a comprehensive management framework. Additionally, raising awareness of irAEs and educating patients, caregivers, and healthcare providers about symptoms that require immediate medical intervention is crucial. The high number of unscheduled visits in our study emphasizes the need for better monitoring and patient education. Structured monitoring programs focused on early detection of irAEs, particularly in the first few months of ICI therapy, can help identify symptoms before they escalate. Telemedicine and regular follow-ups may also reduce emergency visits by providing timely interventions [20–22]. Educating patients and caregivers to recognize early symptoms and seek prompt medical attention is essential to prevent unscheduled hospitalizations [23, 24].

Early detection of irAEs requires not only routine testing, but also a comprehensive and timely assessment focused on the patient's chief complaint. In our study, dyspnea was associated with respiratory symptoms and diarrhea with gastrointestinal toxicity. However, symptoms such as fatigue, anorexia, and vomiting did not show a strong association with specific irAEs. This reflects the non-specific nature of these symptoms and their common occurrence with some irAEs. Fatigue and malaise in cancer patients are multifactorial, caused by both malignancy and chemotherapy, with an incidence of over 60% [25, 26] This reflects the complexity of irAE management and suggests that such symptoms should be carefully monitored, and further diagnostic evaluation is needed to determine the cause. The high rate of emergency presentations in this study among patients presenting with fatigue and fever also suggests the need for patient education to encourage early consultation. Although this study focused on irAE, distinguishing irAE from cancer progression or comorbidity remains a challenge in clinical practice. Early recognition and management can significantly improve patient outcomes and reduce the risk of serious adverse events [27].

This study included 96 patients diagnosed with irAEs requiring emergency hospitalization. Of these, 16 patients with endocrine disorders were excluded from the analysis due to differences in treatment compared to other irAEs. Additionally, patients admitted solely for further evaluation or for reasons unrelated to irAE treatment were excluded from the primary analysis. This ensured the focus remained on patients who required emergency hospitalization for irAE treatment. Among the remaining 80 patients, 62 received corticosteroids, the primary treatment for irAEs, although some patients received antibiotics or alternative therapies, and others improved spontaneously. This discrepancy between guideline-recommended treatment and real-world clinical practice is a notable finding of this study. Conversely, eight patients died due to irAEs, underscoring the potential impact of appropriate irAE management on patient prognosis. Although not all irAEs requiring hospitalization are severe, some require immediate intervention, emphasizing the need for rapid escalation protocols in irAE management. In this study, 34.3% of patients requiring emergency hospitalization for irAEs resumed ICI therapy. Although ICI therapy is crucial in cancer treatment, the risk of recurrent or worsening irAEs remains concerning. Previous reports indicate that approximately 30% of patients experience irAE recurrence [28], suggesting that restarting ICI therapy should be carefully weighed against the potential risks and benefits.

This study has inherent limitations. Its retrospective design depended on the accuracy and completeness of medical records, which may have introduced bias. Furthermore, the study was conducted at a single institution, limiting generalizability to broader populations or settings. Although the sample size was substantial, rare irAEs may have been underrepresented. Future studies should aim to incorporate more comprehensive data collection methods, including standardized grading of symptoms and clarification of overlapping clinical presentations, to provide a more detailed understanding of irAEs. Another limitation is the lack of a direct comparison between patients requiring emergency hospitalization due to irAEs and those who experienced irAEs but did not require hospitalization. While this study focuses on patients who required emergency intervention, a broader comparison with non-hospitalized patients could provide additional insights into the overall management of irAEs. Future research should explore this comparison to enhance understanding of the full spectrum of irAE severity and its management during ICI therapy. Finally, the retrospective nature of the study meant that medical records did not consistently document the severity of irAEs, limiting our ability to fully analyze symptom severity. While the Spearman rank correlation identified associations, it did not establish causality, necessitating prospective investigations to confirm and elucidate these relationships.

## Conclusions

The results of this study emphasize the importance of early detection and management of irAEs, particularly during the first 3 months of ICI therapy, when the risk of severe adverse events is highest. Healthcare providers should focus on monitoring key symptoms such as dyspnea, fatigue, and gastrointestinal disturbances, which were frequently associated with emergency hospitalizations in our cohort. Ensuring that patients and caregivers are well-informed about these symptoms can facilitate earlier intervention, potentially reducing the need for emergency care. These findings underscore the need for vigilant monitoring during critical periods of ICI therapy to improve patient outcomes and to ensure the safe administration of ICI treatments.

## Supplementary Information

Supplementary Material 1.

## Data Availability

The datasets used and/or analyzed in this study are available from the corresponding author upon reasonable request.

## Abbreviations

ICI: Immune checkpoint inhibitor
irAE: Immune-related adverse event
