# Supplementary material for: Clinical characteristics of patients requiring emergency hospitalization due to immune-related adverse events: a retrospective study
Source: J Pharm Health Care Sci. 2024 Dec 18;10:78. doi: 10.1186/s40780-024-00400-7 (PMC11653787; doi:10.1186/s40780-024-00400-7)
Supplement: Supplementary file 1 — Supplementary Material 1. [file 40780_2024_400_MOESM1_ESM.docx]

**Supplementary Material**

**Supplementary Material 1**: The interval to emergency hospitalization by diagnosis of irAE **Supplementary Material 2**: Statistical analysis of chief complaints and diagnoses

**Supplementary Material 3**: Detailed information in posthospitalization outcomes.

Supplementary Material 1. The interval to emergency hospitalization by diagnosis of irAE

This supplementary material presents data on the time interval between the start of ICI treatment and emergency admission to hospital, broken down by diagnosis of irAEs in addition to all patients.

Vertical axis; diagnosis of irAEs

Horizontal axis; time from start of ICI treatment to emergency admission (weeks).

Abbreviations

irAEs; immune-related adverse event, Gastrointestinal; Gastrointestinal disorders, Pulmonary; Pulmonary disorders, Endocrine; Endocrine disorders, Musculoskeletal; Musculoskeletal disorders, Nephrotoxicity; Nephrotoxicity disorders, Neurotoxicity; Neurotoxicity disorders, Cardiovascular; Cardiovascular disorders.

Supplementary Material 2. Statistical Analysis of chief complaints and diagnoses

This supplementary material presents the statistical correlation between chief complaints and irAE diagnoses at the time of emergency hospitalization.

Statistical analysis: Spearman rank correlation coefficient (Rs) and two-sided P values.

Abbreviations

irAEs, immune-related adverse event; Rs, Spearman's rank correlation coefficient.

**Supplementary Material 3**: Detailed information in posthospitalization outcomes.

This supplementary material presents results for posthospitalization outcomes by ICIt treatment regimen and irAEs diagnosis.

Statistical analysis:

Statistical analysis was performed using a chi-squared test of independence to evaluate the association between treatment regimens, irAE diagnoses, and patient outcomes.

Abbreviations

ICI, Immune Checkpoint Inhibitor, irAEs, immune-related adverse event;
